# Research on dynamic creep strain and settlement prediction under the subway vibration loading

**DOI:** 10.1186/s40064-016-2707-2

**Published:** 2016-08-03

**Authors:** Junhui Luo, Linchang Miao

**Affiliations:** Institute of Geotechnical Engineering of Southeast University, Nanjing, 210096 China

**Keywords:** Dynamic characteristics, Dynamic triaxial test, Dynamic shear module, Dynamic creep strain, Settlement

## Abstract

This research aims to explore the dynamic characteristics and settlement prediction of soft soil. Accordingly, the dynamic shear modulus formula considering the vibration frequency was utilized and the dynamic triaxial test conducted to verify the validity of the formula. Subsequently, the formula was applied to the dynamic creep strain function, with the factors influencing the improved dynamic creep strain curve of soft soil being analyzed. Meanwhile, the variation law of dynamic stress with sampling depth was obtained through the finite element simulation of subway foundation. Furthermore, the improved dynamic creep strain curve of soil layer was determined based on the dynamic stress. Thereafter, it could to estimate the long-term settlement under subway vibration loading by norms. The results revealed that the dynamic shear modulus formula is straightforward and practical in terms of its application to the vibration frequency. The values predicted using the improved dynamic creep strain formula closed to the experimental values, whilst the estimating settlement closed to the measured values obtained in the field test.

## Background

With the rapid development of the nation’s economy, subways have become an important traffic reduction strategy in congested urban areas in China (Guo et al. 2012). Nevertheless, subway vibration loading may lead to the non-uniform settlement on subway foundation. Furthermore, it may cause the cracking of tunnel lining and structural cracking. Hence the surrounding environment is adversely affected by vibration (Luo et al. 2015). Accordingly, it is necessary to estimate the settlement during operation, and it represents a key research topic in the design of urban mass transit.

The dynamic modulus is an important parameter used to evaluate the dynamic characteristics of the soil. It is primarily divided into three types, namely the field shear wave test, empirical calculating method and the estimation method (Kyung and Yoo 2014). Nonetheless, the relationship between dynamic modulus and dynamic creep strain is not to establish. Meanwhile, the systematic methods of settlement estimation for evaluating the dynamic characteristics of subway foundation cannot be established.

Numerous research projects have been conducted on the dynamic creep strain function. For instance, Monismith and Ogawan (1975) presented an exponential empirical formula of time-dependent dynamic strain formula. Meanwhile, Li and Selig (1996); Chai and Miura (2002) proposed an improved model based on Monismith’s theory, implementing the settlement prediction. Nonetheless, none of them evaluated the dynamic characteristics of soil. The Singh–Mitchell exponential empirical formulas creep strain model (Singh and Mitchell 1968) evaluated the engineering characteristics of soil. This model can be used to estimate the range from 20 to 80 % shear stress level. However, when the shear stress level is equal to zero, the strain estimation will be less than zero error results. In an attempt to address this issue, Mesri modified the Singh–Mitchell model to devise the Mesri empirical formulas model (Mesri et al. 1981; Kondner 1963). Subsequently, it can be computed arbitrary shear stress level. Accordingly, it is suitable for the analysis of low stress level, including subway vibration loading.

Meanwhile, the dynamic stress amplitude is required for estimating the settlement of subway foundation, and the finite element method is commonly utilized (Olsson and Kallsner 2015). Metrikine and Vrouwenvelder (2000); Paulo et al. (2015) devised the subway finite element model to determine the dynamic stress and analyzed dynamic response under vibration loading. In order to analyze the dynamic characteristics of the subway foundation soil along different direction during operation, Forrest and Hunt (2006) created the 3D finite element model to estimate the settlement.

As such, this research focuses on the dynamic characteristics of soft soil and the settlement prediction. The dynamic shear modulus formula considering vibration frequency was established, then the proposed formula was introduced into the dynamic creep strain function. Subsequently, the dynamic stress of the subway foundation was obtained through finite element simulation. Then the improved dynamic creep strain curve was determined by the dynamic stress. Thereby, the settlement of the subway foundation was estimated. This study would be helpful to illuminate new theoretical soft soil research on dynamic characteristics and settlement prediction. It also has profound guiding significance in subway engineering practices.

## Experimental study on dynamic shear modulus

### Analysis of dynamic triaxial test

Shield segment can be affected by subway vibration loading during operations (Shen et al. 2014). In order to simulate the subway vibration loading on soil, the dynamic triaxial test was performed.

**Fig. 1:**
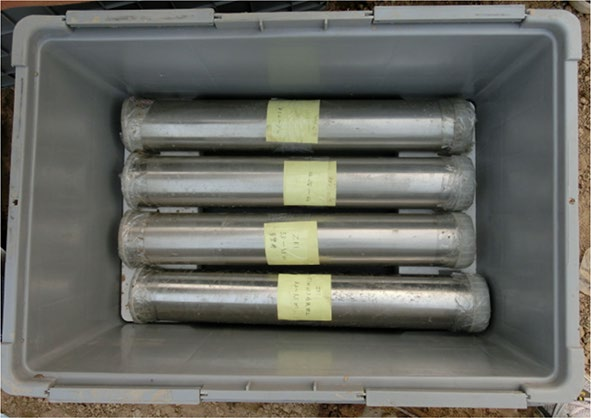
Thin soil sampler

Firstly, using the thin soil sampler (as shown in Fig. 1) to collect the in-situ undisturbed soil. The location of the obtained undisturbed soil was in the Hexi area of Nanjing, China, Metro Line 2 of Nanjing crossed through the area. In order to facilitate the test, the undisturbed soil samples were made of cylinder sample cylinders that were 38 mm in diameter and 75 mm in height. Meanwhile, the researcher ensured that the sample preparation was performed carefully to ensure the structure of undisturbed soil samples were not disturbed.

Thereafter, the physical and mechanical indexes of undisturbed soil were obtained through normal laboratory experiments, as detailed in Table 1.

**Table 1:** Physical and mechanical properties of soft clay

Natural water content w/%	Specific gravity *d*_*s*_	Density ρ/g/cm^3^	Void ratio e	Plastic index *I*_*p*_
37.3–45	2.72	1.877	1.14	17

**Fig. 2:**
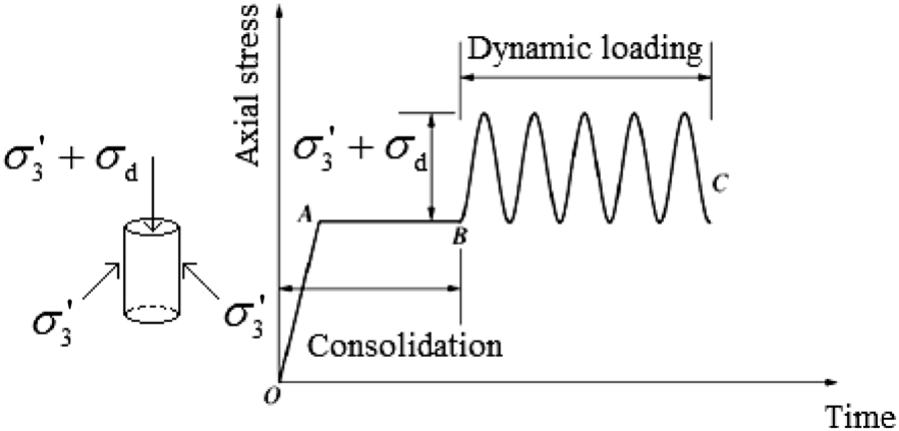
Loading mode

Studies have shown that the subway loading produce a vibration waveform which is similar to the sine wave (Rucker 1977). Based on this, during the dynamic triaxial test, the loading mode used in the test is shown in Fig. 2. Where $\sigma_{3}^{'}$ is effective confining pressure whereas $\sigma_{3}^{'}+\sigma_{d}$ is the total vertical effective dynamic stress. The dynamic triaxial test was performed with the undisturbed cylinders samples (left-hand section of Fig. 2). The test process is shown on the right-hand side of Fig. 2. Firstly, the saturation stage is carried out (0–A), before the sample is subjected to the effective confining pressure $\sigma_{3}^{'}$ during the isotropic consolidation process (A–B). Thereafter, the vertical effective load is loaded during the vibration stage (B–C) (Zhang et al. 2013). For instance, when the effective confining pressure $\sigma_{3}^{'}$ was 75 kPa, and the dynamic stress *σ*_d_ was 8.3 kPa, and the median value of dynamic stress was 4.15 kPa. Hence, the total vertical effective dynamic stress $\sigma_{3}^{'}+\sigma_{d}$ was 83.3 kPa.

**Fig. 3:**
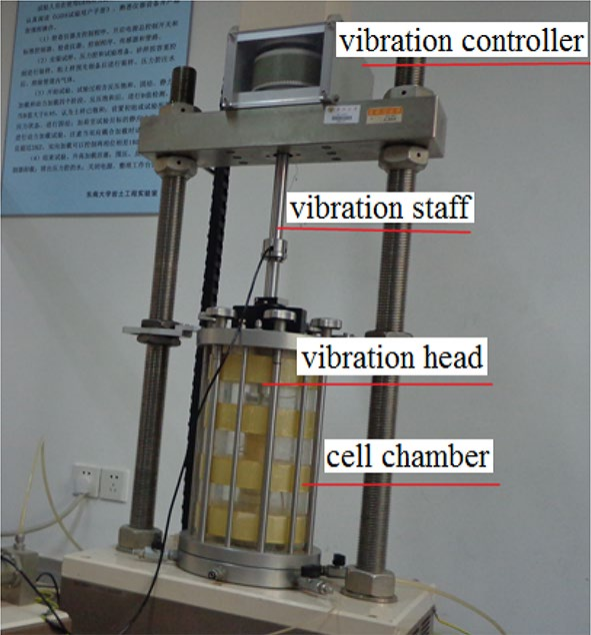
Dynamic triaxial apparatus

In Fig. 3, it was the dynamic triaxial apparatus. During operation, the vibration staff (controlled by the vibration controller) driver the oscillating vibration head up and down so that vibration loading was applied to the soil in the cell chamber.

Meanwhile, with the different in-situ sampling depth of the soil, the confining pressure was different. According to the effective unit weight of soil and sampling depth required to determine the corresponding effective confining pressure during the dynamic triaxial test.

Based on the hypothesis of isotropy, the effective confining pressure $\sigma_{3}^{'}= d \cdot \gamma^{'}$ is conducted during the dynamic triaxial test. Where *d* is the sampling depth of undisturbed soil, $\gamma ^{'}= \rho^{'}\cdot g$ the effective unit weight of soil, $\rho^{'}$ the effective density of soil and *g* the acceleration of gravity.

In this research, the effective confining pressure was 75 and 150 kPa, respectively. It used a half Sine wave load (Rucker 1977).

The dynamic loads were 10, 15, and 20 N, (8.3, 12.5, 16.7 kPa) respectively;

According to the existing literature (Ng et al. 2013), the vibration loading of subways was predominantly in the low frequency range, so the frequency of the test was set as: 1, 0.5 and 2 Hz respectively. Moreover, it established 10,000 vibration times.

### Experimental estimation of dynamic shear modulus

The dynamic shear modulus is $G_{d} = {{\tau_{\text{d}} } \mathord{\left/ {\vphantom {{\tau_{\text{d}} } {\gamma_{\text{d}} }}} \right. \kern-0pt} {\gamma_{\text{d}} }}$ under vibration loading. Where $\tau_{\text{d}}$ is dynamic shear stress and $\gamma_{\text{d}}$ is dynamic shear strain, and they are the test results automatically obtained by the dynamic triaxial apparatus.

The case of dynamic loading was F_d_ = 20 N($\sigma_{\text{d}} = 16.7\,{\text{kPa}}$) f = 1, 0.5 and 2 Hz respectively, and the strain range of this study is 5 × 10^−4^ to 5 × 10^−2^. The corresponding dynamic shear modulus when the strain equals to 0 are shown in Table 2.

**Table 2:** The test values of dynamic shear modulus

Effective confining pressure/kPa	Frequency/Hz	Dynamic shear modulus/MPa
75	0.5	26
	1	33
	2	39
150	0.5	41
	1	45
	2	54

Under the same conditions, by increasing the effective confining pressure, the soil was compacted, the void ratio of soil decreased, the dynamic deformation reduced and the dynamic shear modulus increased. Thereafter, by increasing the vibration frequency, the time was shorter and the deformation of soil decreased, whilst corresponding dynamic shear modulus increased when the strain equals to 0.

### Empirical formula of the dynamic shear modulus

When lacking the necessary test equipment, the empirical method based on the physical and mechanical properties can accurately estimate the dynamic shear modulus (Luo et al. 2015).

**Fig. 4:**
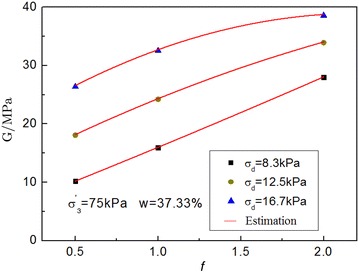
Relation curves between the dynamic shear modulus and frequency

In order to illustrate the influence of frequency, according to the existing literature (Ng et al. 2013; Liu 2013; Zhai and Liu 2005), the frequency was changed with the varied speed when the subway run. The effect of speed on subway vibration loading under the same sampling depth mainly changes by frequency. Hence, in this case, the vibration frequency affected the dynamic stress *σ*_d_. Therefore, the frequency of the soft soil was a pivotal factor. The relationship between frequency and dynamic shear modulus can be expressed by a hyperbolic function, as shown in Fig. 4. Based on Kagawa (1992) empirical formula, the improved formula for frequency was proposed.

Kgawa’s empirical formula is as follows:

1 $$G = \frac{{358 - 3.8I_{p} }}{0.4 + 0.7e}\sigma_{3}^{'}$$

where *I*_*p*_ is plastic index, $\sigma_{3}^{'}$ is effective confining pressure and *e* void ratio of soil.

According to the reference (Sas et al. 2015), the dynamic shear modulus improved formula was analyzed by square regression:

2 $$G_{d} = \frac{{358 - 3.8I_{p} }}{0.4 + 0.7e}\sigma_{3}^{'}\cdot \left( {a_{1} \cdot f^{2} + a_{2} \cdot f + a_{3} } \right)$$

where the unit of  *f* is dimensionless, which is frequency; *I*_*p*_ = *17* is plastic index, *e* = 1.14 in Table 1; a_1_, a_2_ and a_3_ are calculated by square regression analysis, as shown in Table 3.

**Table 3:** Parameters of the dynamic shear modulus formula

Effective confining pressure/kPa	*f*	Coefficients			Correlation values
		*a*_*1*_	*a*_*2*_	*a*_*3*_	
75	0.5	−4.16	18.52	18.35	0.993
	1	−1.74	14.91	11.15	0.994
	2	0.31	11.12	4.58	0.991
150	0.5	−3.9	20.5	5.8	0.996
	1	−5.3	20.1	23.3	0.993
	2	0.22	8.14	37.4	0.992

Therefore, based on Kgawa’s model, an improved model considering the frequency is established for estimating the dynamic shear modulus and dynamic creep strain.

## Estimation and analysis of dynamic creep strain

### Estimation of dynamic creep strain

Mesri creep model (Mesri et al. 1981) was calculated for the dynamic creep strain time history curve. When the time last long under dynamic loading, the soft soil would occur creep strain, so it is called the dynamic creep strain. The Singh-Mitchell model (Singh and Mitchell 1968) only describes the characteristics of soil under shear stress in the range of 20–80 %. When the shear stress level was zero, the estimation of strain would be less than zero. Due to these shortcomings, Mesri improved the Singh–Mitchell creep model. Mesri model can be used to calculate the creep strain of the soil under arbitrary shear stress levels. The shear stress level was no longer limited to the range of 20–80 %, instead including all the stress levels (0–100 %), with the deduction as follows.

According to the Mesri formula:

3 $$\varepsilon_{s}^{t} = \frac{2}{{(E_{d} /S_{u} )}}\frac{{\overline{D} }}{{1 - (R_{f} ) \cdot \overline{D} }}\left[ {\frac{{(t)_{i} }}{t}} \right]^{m}$$

where m are the model parameters, (t)_*i*_ the unit time, *t* the time, $\overline{D} = {{\left( {\sigma_{1} - \sigma_{3} } \right)} \mathord{\left/ {\vphantom {{\left( {\sigma_{1} - \sigma_{3} } \right)} {\left( {\sigma_{1} - \sigma_{3} } \right)_{\text{f}} }}} \right. \kern-0pt} {\left( {\sigma_{1} - \sigma_{3} } \right)_{\text{f}} }}$ the shear stress level and $S_{\text{u}} = {{\left( {\sigma_{1} - \sigma_{3} } \right)_{\text{f}} } \mathord{\left/ {\vphantom {{\left( {\sigma_{1} - \sigma_{3} } \right)_{\text{f}} } 2}} \right. \kern-0pt} 2}$ the undrained shear strength. The dynamic modulus $E_{d} = G_{d} \cdot 2(1 + \mu )$ (Hardin and Drnevich 1972) is calculated by Formula (2). $R_{\text{f}} = {{\left( {\sigma_{1} - \sigma_{3} } \right)_{\text{f}} } \mathord{\left/ {\vphantom {{\left( {\sigma_{1} - \sigma_{3} } \right)_{\text{f}} } {\left( {\sigma_{1} - \sigma_{3} } \right)_{ult} }}} \right. \kern-0pt} {\left( {\sigma_{1} - \sigma_{3} } \right)_{ult} }}$ the damage ratio.

**Fig. 5:**
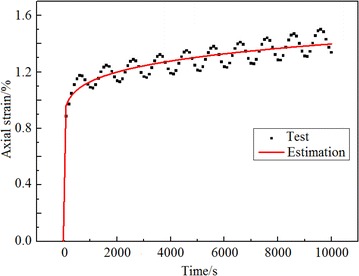
Dynamic strain time history curve

According to Formula (3), the dynamic creep strain time history curve was calculated, thereby determining the parameter *m*. As shown in Fig. 5, owing to the action of the vibration loading, the corresponding test value of dynamic creep strain time history curve appeared the pulse phenomenon.

The parameters of this established dynamic creep strain model under different factors by Formula (3) were in Table 4.

**Table 4:** Parameters of dynamic creep strain model

Natural water content w/%	Dynamic stress amplitude σ_d_/kPa	Effective confining pressure $\sigma_{3}^{'}$/kPa	Frequency *f/*Hz	*E*_*d*_/*S*_*u*_	*R*_f_	*m*	Correlation values
37	8.33	75	0.5	1025	0.72	0.933	0.978
			1.0	1614	0.75	0.948	0.981
			2.0	2839	0.77	0.903	0.913
37	8.33	150	0.5	947	0.64	0.947	0.827
			1.0	1407	0.67	0.903	0.913
			2.0	1958	0.66	0.945	0.870
37	12.5	75	0.5	1836	0.71	0.925	0.965
			1.0	2454	0.72	0.934	0.933
			2.0	3450	0.74	0.926	0.935
37	12.5	150	0.5	2031	0. 57	0.909	0.975
			1.0	2424	0. 64	0.896	0.984
			2.0	2662	0. 61	0.918	0.906
37	16.67	75	0.5	2679	0.72	0.938	0.884
			1.0	3296	0.70	0.936	0.979
			2.0	3914	0.74	0.865	0.913
37	16.67	150	0.5	2600	0.52	0.851	0.998
			1.0	2864	0.56	0.923	0.965
			2.0	3420	0.57	0.847	0.993
45	16.67	75	0.5	1257	0.69	0.972	0.919
			1.0	1786	0.73	0.941	0.826
			2.0	2568	0.71	0.918	0.889
45	16.67	150	0.5	1300	0.51	0.945	0.870
			1.0	1527	0.55	0.903	0.913
			2.0	1773	0.53	0.934	0.933

### Comparative analysis of dynamic creep strain

According to the above formula, the influence of dynamic stress, frequency, effective confining pressure and natural water content on the dynamic creep strain of soil was analyzed.

### The influence of dynamic stress

**Fig. 6:**
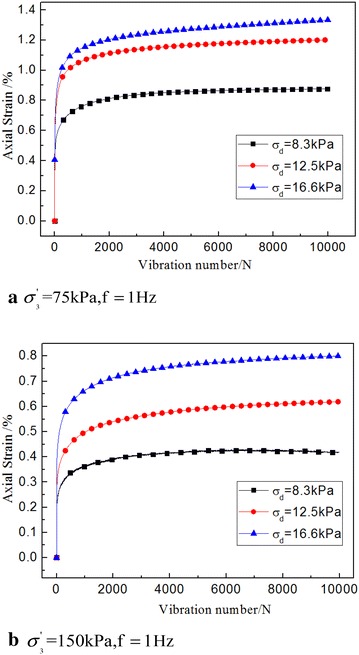
Dynamic strain time history curve under different dynamic stress amplitude

In Fig. 6, under the same condition, the vibration loading was greater, the larger the vibration energy was, thus the soil had greater kinetic energy, as was the corresponding dynamic creep strain.

However, although the dynamic stress was in a certain linear proportion (8.3, 12.5 and 16.6 kPa), the corresponding dynamic creep strain was not similar to the linear law. It revealed that the soil was a non-linear material.

### The influence of frequency

**Fig. 7:**
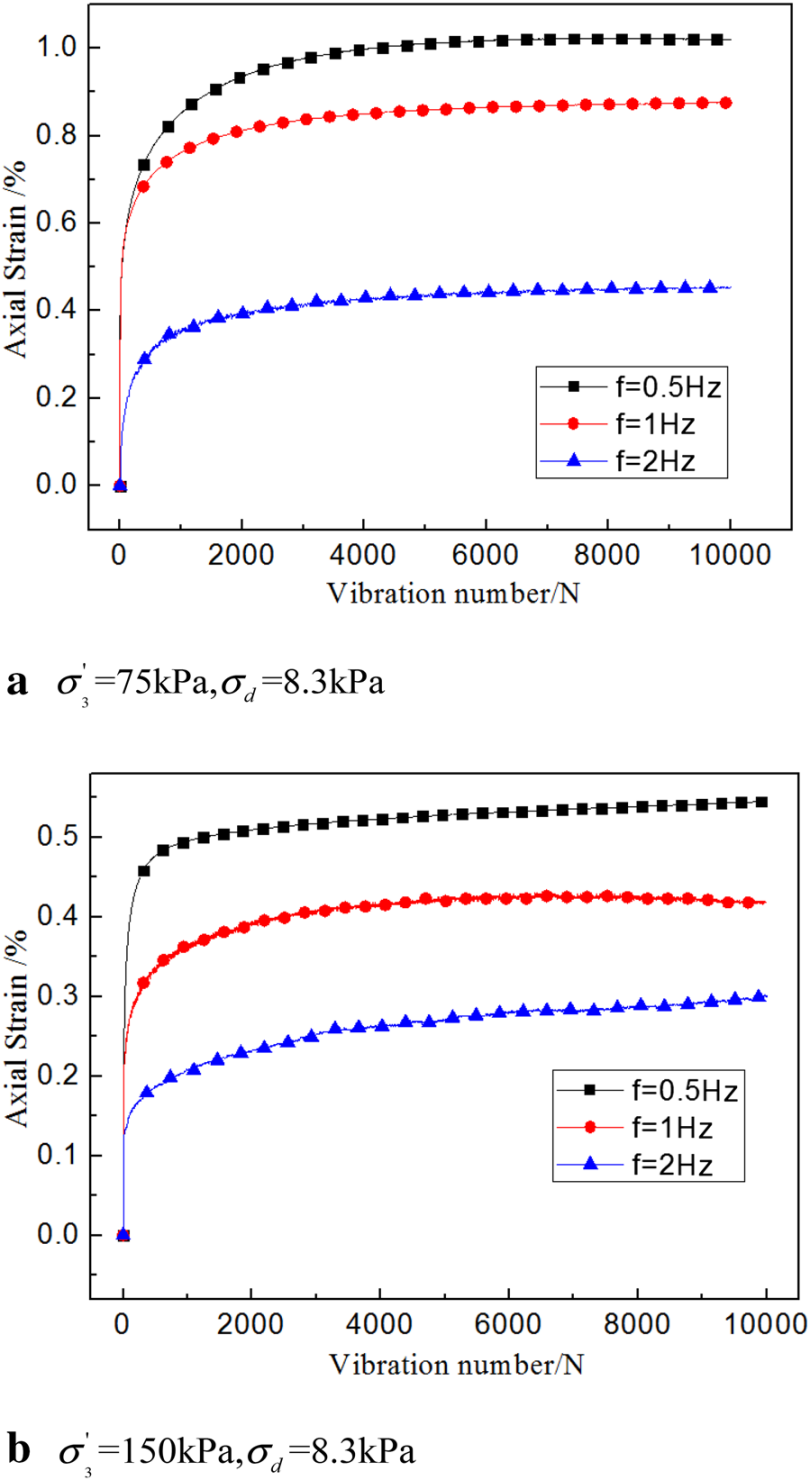
Dynamic strain time history curve under different frequencies

The essence of the effect of frequency on the dynamic creep strain was the different duration of dynamic load. Therefore, the higher the frequency, the faster the load changed, the shorter the action lasted, the less energy that was transferred and the smaller the dynamic strain was. Such findings are shown by Fig. 7.

### The influence of effective confining pressure

**Fig. 8:**
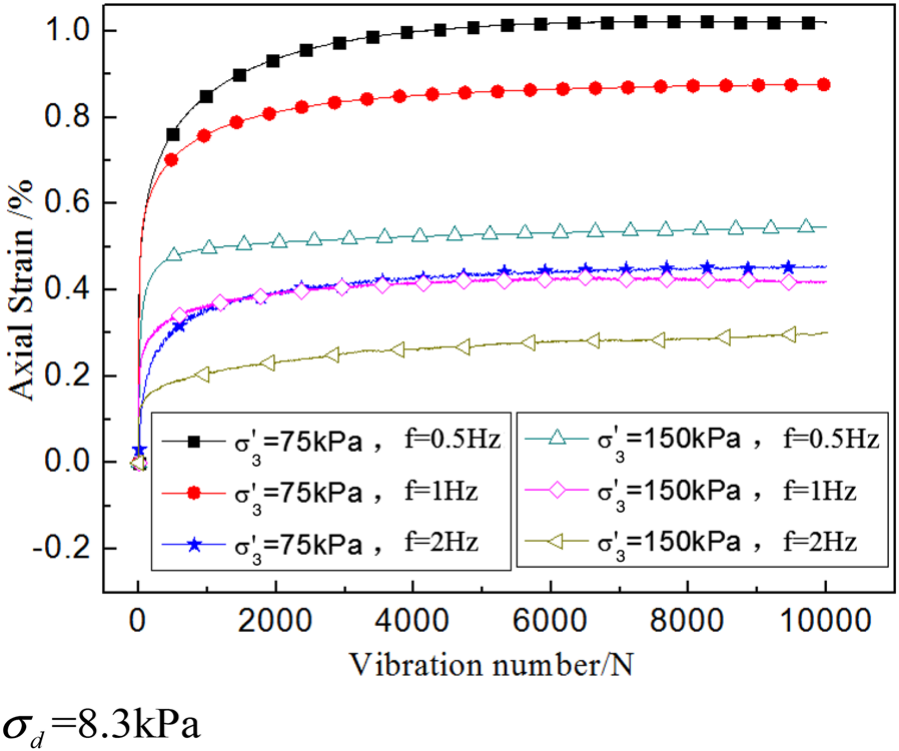
Dynamic creep strain time history curve under different confining pressure

The influence of effective confining pressure on the dynamic strain is due to the difference in density degree. When the dynamic triaxial test was carried out, different levels of effective confining pressure were set. In Fig. 8, according to the sampling depth and density of the undisturbed soil, the greater sampling depth, the lower the dynamic creep strain of the soil.

### The influence of natural water content

**Fig. 9:**
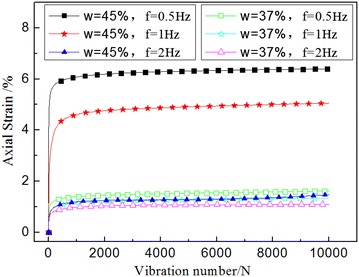
Dynamic creep strain time history curve under different natural water content

Natural water content was a parameter of physical and mechanical properties, measuring the moisture content of soil in the natural state. In Fig. 9, the effect on dynamic creep strain reflected that when the natural water was large, the film layer of soil particles was also thick, particle spacing was bigger, the attraction between soil particles decreased, and were prone to dislocation. Therefore, the larger natural water content would produce a greater dynamic creep strain deformation under vibration loading.

## Estimation of dynamic stress under vibration loading

Subway foundation soil would occur settlement during operation, which can be estimated by establishing the improved dynamic creep strain formula. The corresponding dynamic stress of each soil layer should be obtained before estimating the settlement (Ju 2009).

According to the design of dynamic stress formula:

4 $$\sigma_{{{\text{d}}_{ 0} }} = 0.26{\text{P(1}} \pm 0. 0 4 {\text{V)}}$$

where $\sigma_{{{\text{d}}_{ 0} }}$ is dynamic stress acting on the track bed, P the subway train weight and V subway speed, recorded at 80 km/h.

It obtained $\sigma_{{{\text{d}}_{ 0} }}$ = 69 kPa, which was then substituted into the subway finite element model (Ju 2009). The varied additional stress $\sigma_{{{\text{d}}_{i} }}$ with sampling depth can be obtained at the bottom of the tunnel axis by the finite clement calculation, then determined the corresponding dynamic creep strain under first cyclic load. Subsequently, the following was settlement estimation. The case study was Yuantong station of Nanjing subway tunnel, which had a distance of 9–12 m from ground, thereby establishing the two-dimensional model of the subway.

### Case analysis

**Fig. 10:**
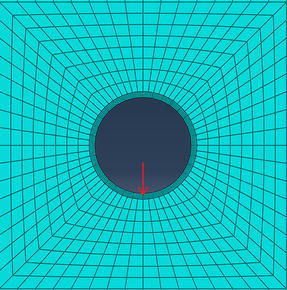
The simulation of finite element

The subway inner diameter is 5.6 m, the outside diameter 6.2 m. The lining thickness is 0.35 m, the lateral width 100 m. The center of the tunnel from ground is 9 m, the center of the tunnel from the bottom 18 m and the height 50 m. As shown in Fig. 10, the subway vibration load acted on the center of the tunnel.

### Constitutive model parameters

In order to obtain the constitutive model parameters of the soft soil on the subway foundation, the parameters were obtained through dynamic and static triaxial test as shown in Table 5.

**Table 5:** Parameters of constitutive model

*E*d/MPa	*c*/kPa	φ/°
38	10	11

### Estimation of dynamic stress with sampling depth

**Fig. 11:**
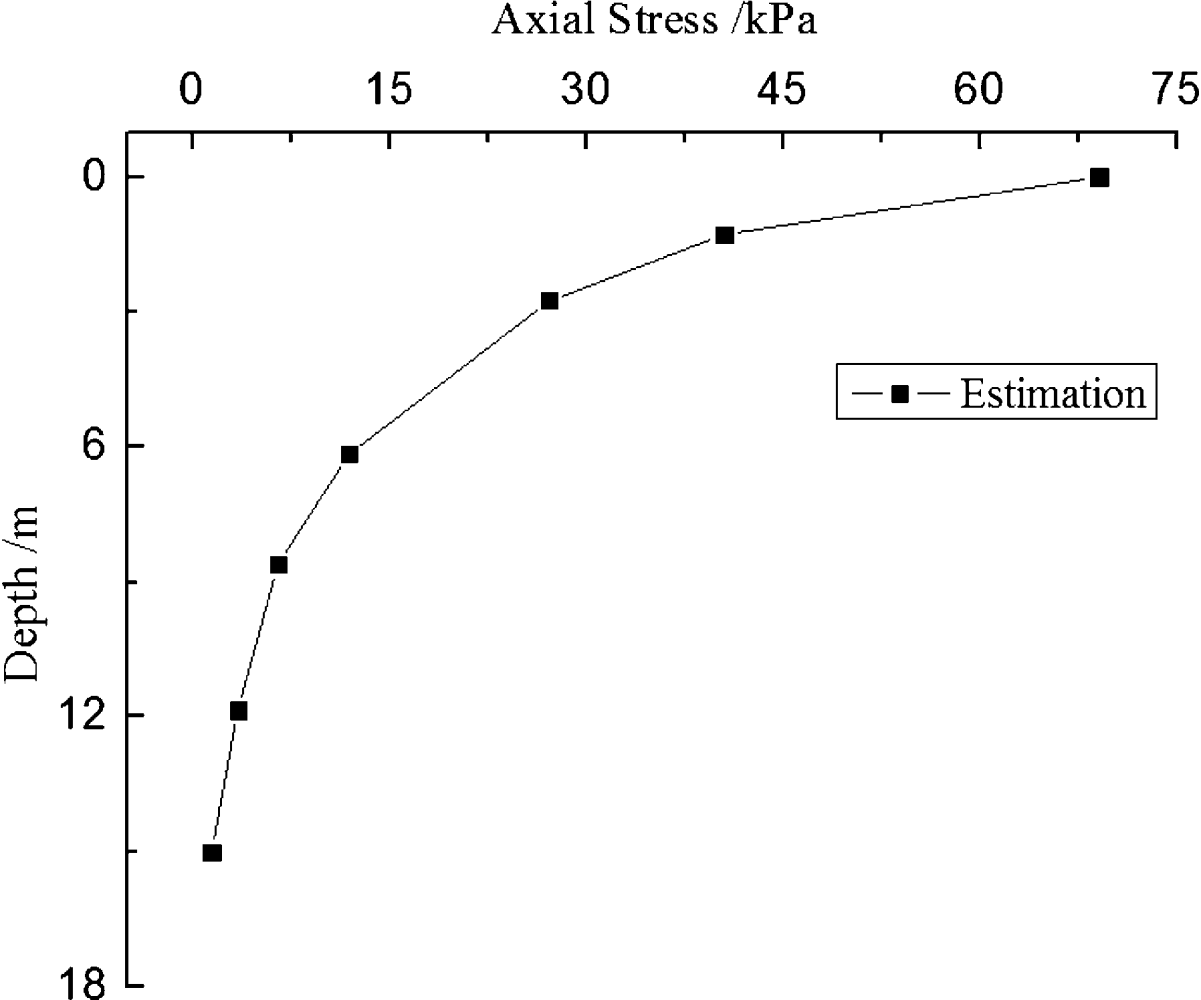
Additional stress with sampling depth in the bottom of the tunnel

It obtained $\sigma_{{{\text{d}}_{i} }}$ the additional stress curve with sampling depth illustrated in Fig. 11.

## Settlement estimation

**Steps**

The improved dynamic creep strain in Formula 3 and the additional stress $\sigma_{{{\text{d}}_{i} }}$ with sampling depth are used to estimate the settlement of the subway foundation, the main calculation steps are as follows:

1. Firstly, the dynamic stress amplitude acting on the track bed was determined, and the dynamic stress at the bottom of 0 m sampling depth was $\sigma_{{{\text{d}}_{ 0} }}$ = 69 kPa.
2. The above step results were substituted into the subway finite element model, then the additional stress $\sigma_{{{\text{d}}_{i} }}$ with sampling depth was estimated.
3. According to the influence of the vibration response, thickness of the vertical deformation could be determined, and the vertical layers were divided by norms.
4. The vertical strain deformation of each layer was estimated by the improved dynamic creep strain function.
5. The settlement of subway soil foundation was estimated by the layer-wise summation method of norm, expressed as (Jin 2004):

9 $$\Delta H = \sum\limits_{{{\text{i}} = 1}}^{\text{n}} {\varepsilon_{zi} } {\text{H}}_{i}$$

where $\varepsilon_{zi}$ is the vertical accumulated creep strain of the *i* layer; H_i_ the height of the *i* layer and *n* the number of layers in the scope of influence distance on the bottom of the tunnel.

### Comparative analysis

**Fig. 12:**
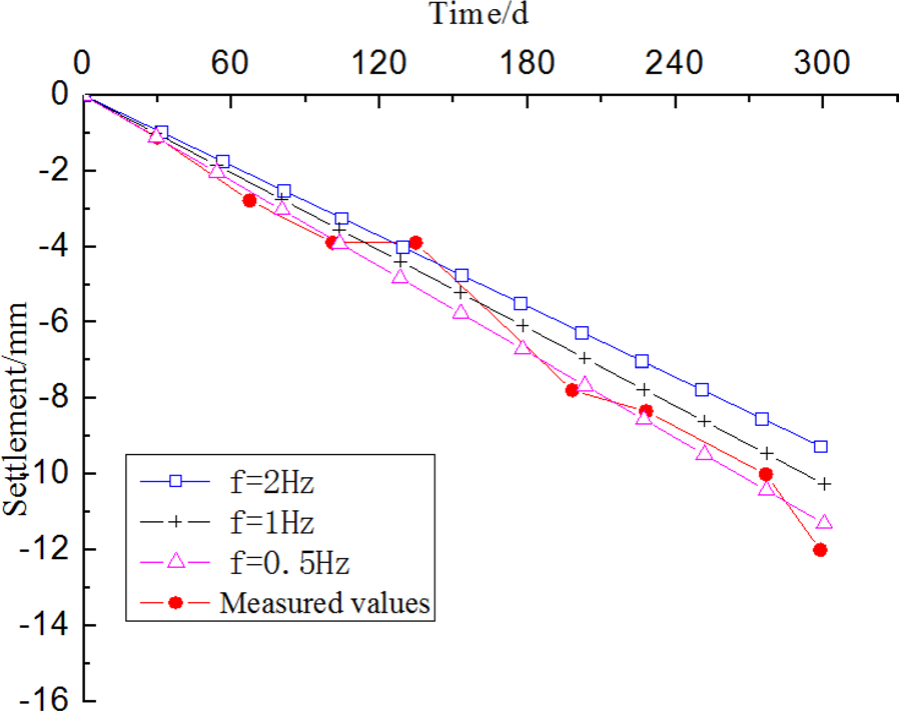
Comparison of the results of settlement between the measured values and the estimation obtained using the finite element method

Based on the above steps, the long-term settlement under the condition of different frequencies (0.5, 1 and 2 Hz) can be estimated. Meanwhile, using the long-term settlement of measured values obtained by the field test data, the results are shown in Fig. 12. It reveals that the estimated values closed to the measured values obtained in the field test.

## Conclusion

The following conclusions were obtained by analyzing the dynamic characteristics and long-term settlement of the soil under vibration loading. The methods of laboratory testing, theoretical analysis and numerical simulation were using.

1. The case of dynamic load by dynamic triaxial test was F_d_ = 20 N(16.7 kPa), the frequency f = 1, 0.5, 2 Hz respectively. And the case test results showed that under the same conditions, by increasing the effective confining pressure, the dynamic deformation reduced and the corresponding dynamic shear modulus increased when the strain equals to 0. Thereafter, by increasing the frequency, the time of load acting on the soil was shorter and the deformation of the soil decreased, whilst the dynamic shear modulus increased.
2. Based on the Kgawa model, the improved dynamic shear modulus formula which considered the frequency was established. The results demonstrate that the correlation values of estimation calculated by the improved dynamic shear modulus formula are between 0.991–0.996, thereby verifying the validity of the improved formula.
3. Subsequently, the improved formula was applied to the dynamic creep strain model function. It suggested that the correlation values of the estimated values generated by the improved dynamic creep strain formula are between 0.826–0.998.
4. Meanwhile, the effects of amplitude, frequency, effective confining pressure and natural water content on the dynamic creep strain were analyzed. It showed that the larger the vibration amplitude, the greater the dynamic creep strain, whilst the larger the frequency, the smaller the dynamic creep strain. Likewise, the larger the effective confining pressure, the smaller the dynamic creep strain, and the larger the natural water content, the greater the dynamic creep strain.
5. The speed of case subway train recorded at 80 km/h. According to the design of the dynamic stress formula, the dynamic stress acting on the track bed at the bottom of 0 m sampling depth is 69 kPa. Thereafter, the dynamic stress was substituted into the subway finite element simulation to obtain additional stress curve with sampling depth acting on the soil foundation. Based on the improved dynamic creep strain function and additional stress, the long-term settlement was obtained under subway vibration loading by norms. It showed that the estimated values of the long-term settlement under the conditions of different frequencies (0.5, 1 and 2 Hz) closed to the measured values obtained through the field test.

## References

[CR2] Chai JC, Miura N (2002). Traffic-load-induced permanent deformation of road on soft subsoil. J Geotech Geoenviron Eng.

[CR3] Forrest JA, Hunt HEM (2006). A three-dimensional tunnel model for calculation of train-induced ground vibration. J Sound Vib.

[CR4] Guo WW, Xia H, De Roeck G, Liu K (2012). Integral model for train–track –bridge interaction on the Sesia viaduct: dynamic simulation and critical assessment. Comput Struct.

[CR5] Hardin BO, Drnevich VP (1972). Shear modulus and damping in soils: design equations and curves. J Soil Mech Found Div ASCE.

[CR6] Jin B (2004). Dynamic displacements of an infinite beam on a poroelastic half space due to a moving oscillating load. Arch Appl Mech.

[CR7] Ju SH (2009). Finite element investigation of traffic induced vibrations. J Sound Vib.

[CR8] Kagawa T (1992). Moduli and damping factors of soft marine clays. J Geotech Eng.

[CR9] Kondner RL (1963). Hyperbolic stress-strain response: cohesive soils. J Soil Mech Found ASCE.

[CR10] Kyung JS, Yoo B (2014). Rheological properties of azuki bean starch pastes in steady and dynamic shear. Starch-Starke.

[CR11] Li D, Selig ET (1996). Cumulative plastic deformation for fine-grained sub grade soils. J Geotech Eng.

[CR12] Liu F (2013). Long-term settlement of metro in soft ground and its influence on safety.

[CR13] Luo JH, Miao LC, Wang ZX, Shi WB (2015). Modified cam-clay model with dynamic shear modulus under cyclic loads. J VibroEng.

[CR15] Mesri G, Febres CE, Shields DR, Castro A (1981). Shear − stress − strain − time behavior of clays. Geotechnique.

[CR16] Metrikine AV, Vrouwenvelder ACWM (2000). Surface ground vibration due to a moving train in a tunnel: two dimensional model. J Sound Vib.

[CR17] Monismith CL, Ogawan Freeme CR (1975). Permanent deformation characteristics of subsoil due to repeated loading. Transp Res Rec.

[CR18] Ng CWW, Liu GB, Li Q (2013). Investigation of the long-term tunnel settlement mechanisms of the first metro line in Shanghai. Can Geotech J.

[CR19] Olsson A, Kallsner B (2015). Shear modulus of structural timber evaluated by means of dynamic excitation and FE analysis. Mater Struct.

[CR20] Paulo AM, Costa Pedro A, Godinho Luis MC (2015). 2.5D MFS-FEM model for the prediction of vibrations due to underground railway traffic. Eng Struct.

[CR21] Rucker W (1977) Measurement and evaluation of random vibrations. Proceedings of DMSR p 407–421

[CR22] Sas W, Gabrys K, Szymanski A (2015). Effect of time on dynamic shear modulus of selected cohesive soil of one section of express way no. S2 in Warsaw. Acta Geophys.

[CR23] Shen SL, Wu HN, Cui YJ (2014). Long-term settlement behaviour of metro tunnels in the soft deposits of Shanghai. Tunn Undergr Space Tech.

[CR24] Singh A, Mitchell JK (1968). General stress-strain-time function for soils. J Soil Mech ASCE.

[CR25] Zhai H, Liu WN (2005). A study on the low frequency ground response induced by metro train and corresponding vibration reduction measures. Urban Rapin Rail Transit.

[CR26] Zhang JF, Chen JJ, Wang JH (2013). Prediction of tunnel displacement induced by adjacent excavation in soft soil. Tunn Undergr Space Tech.

